# Commentary: The body embarrassed—A commentary on Černis et al. (2026) and Martinez et al. (2026)

**DOI:** 10.1002/jcv2.70166

**Published:** 2026-08-23

**Authors:** Alexandra Moussa‐Tooks, Natasha Chaku

**Affiliations:** ^1^ Department of Psychological and Brain Sciences Indiana University Bloomington Bloomington Indiana USA; ^2^ Program in Neural Science Indiana University Bloomington Bloomington Indiana USA; ^3^ Cognitive Science Program Indiana University Bloomington Bloomington Indiana USA

**Keywords:** anomalous experience, biopsychosocial, hormones, puberty, self‐report

## Abstract

**What's Known?**

- In this 2026 issue of *JCPP Advances*, Černis et al. and Martinez et al. highlight important considerations for the assessment of psychopathology during adolescence. Černis et al. demonstrate that felt sense of anomaly is normative during adolescence while certain anomalous experiences, such as depersonalization, may serve as important transdiagnostic markers for psychopathology risk. Relatedly, Martinez et al. show that informant reports begin to diverge during adolescence, with self‐reported psychopathology endorsed higher than caregiver and teacher reports.

**What's New?**

- Anomalous body experience may be a key mechanism linking hormonal, brain, and social behaviors to psychopathology and for understanding the complex etiology underlying the emergence of clinical disorders.

**What's Relevant?**

- This emerging body of work holds promise for identifying individuals at heightened risk for psychopathology and for developing precise and adaptable interventions, which may occur at the individual or population levels.

It would be naive to argue that bodies at any given moment in a complex culture are understood socially or felt experientially in only one way.

(from The body embarrassed: Drama and the disciplines of shame in early modern England, G. K. Paster (1993))

The transformation of the physical body is key to development. The vagaries of its development, and one's own personal experience of it, are therefore central to phenomenological accounts of the emergence of atypical development, including psychopathology. Dramatic changes in body experience happen across the lifespan but are concentrated in childhood and adolescence coinciding with peak onset of many clinical disorders including anxiety, depression, and psychosis among others. Understanding when these bodily experiences emerge, for whom they become distressing, and why they might persist, may therefore provide key insight into the putative mechanisms linking normative developmental experiences to psychopathology.

Adolescence, marking the developmental period between childhood and adulthood, is characterized by dramatic physical development that precipitates substantial changes in the body and mind (Chaku & Davis‐Kean, 2024). Physically, youth transition through puberty during which they experience growth spurts, skeletal changes such as widening of the pelvis, development of sex organs, and changes in distribution of adipose tissue and muscle. Notably, this growth is uncoordinated, occurring first in the extremities (e.g., hands and feet) and then later in the trunk and torso. These rapid changes in body size and shape require continual updating of body schema (i.e., the sensorimotor representation of the body). Together, physical changes and perception of those changes alter not only the absolute size of the body, but also its proportions, coordination, and internal signals, potentially placing increased demands on the central and peripheral neural systems responsible for monitoring, updating, and integrating bodily signals (Sestito et al., 2017).

For most youth, these body‐based changes and related changes in self‐experience are expected and transient. As published in this 2026 Special Issue of *JCPP Advances*, Černis et al. (2026) show that felt sense of anomaly (FSA) is normative in adolescence. This study investigated self‐reported FSA using the previously established Černis Felt Sense of Anomaly Scale (ČEFSA), which assesses dissociative experiences of self, body, agency, reality, familiarity, connection, and emotion. In their sample of more than 3000 adolescents aged 13–18 years, over 90% of participants endorsed at least one anomalous experience as occurring often or always over a 2‐week period. Specifically, youth most often endorsed an altered sense of agency, consistent with typical adolescent experiences of disconnection from peers and others. The authors also found these endorsements to be consistent at a 1‐month follow‐up, again highlighting the normative nature of these experiences. For some youth, however, benign or ambiguous bodily sensations may be experienced as unusually diffuse within domain (i.e., across many bodily regions) or across domains (i.e., anomalous agency, and body, and self, etc.), persistent (i.e., continuing past the putative end of the physical transition), or intense (i.e., salient, overwhelming, or distressing) (Moussa‐Tooks et al., 2026). Indeed, Černis et al. (2026) found that derealization (i.e., altered sense of familiarity) and anomalous body experience (depersonalization) were endorsed at the lowest rates, suggesting that these specific dissociative experiences may be powerful indicators of atypical development, and thus, pathology and function. Growing work supports this hypothesis, highlighting the links between aberrant bodily experience and psychiatric phenomena, but also broader health challenges (Petersen et al., 2020).

Physical transformation of the body is accompanied by marked changes in its perception. Accordingly, the role of bodily perception in health and wellbeing further highlights the value of self‐report of these perceptual shifts. Indeed, as highlighted in the article by Martinez et al. (2026) published in this current 2026 Special Issue of *JCPP Advances*, psychopathology begins to diverge significantly between informants. Using the longitudinal Adolescent Brain Cognitive Development (ABCD) study, which includes nearly 12,000 youth, Martinez et al. (2026) report that youth consistently rate their own psychopathology higher than caregivers or instructors. This discrepancy grows over adolescence; spans internalizing, externalizing, and inattention symptoms; and varies depending on demographics characteristics of the youth and reporter. Thus, the primary driver of psychopathology is likely the individual's experience (or interpretation of how others perceive them). Accordingly, in relation to measurement, informant contributions change from childhood to adulthood. Regarding bias, Martinez et al. (2026) note that factors like caregiver education and race contributed to reporting differences. Similarly, discrepancies were more pronounced for youth who experienced earlier puberty (Martinez et al., 2026). This finding is of particular note because it reflects the stark importance of informant (self vs. other) differences when reporting on internal experience and potential biases from external factors that influence informant reports. Extending these findings to those by Černis et al. (2026), anomalous experience of the body and self are, by nature, subjective internal experiences. The added value of adult reports on child behaviors begin to shift, more so reflecting observed impairment, functional change, and even bias. That is, although parents, teachers, and other adults may provide important information about changes in behavior, impairment, and functioning, they have limited access to the salient internal experiences that contribute to psychopathology. Taken together, the ability for a guardian or instructor to capture the driving mechanisms of psychopathology may be limited in adolescence.

The work by Martinez et al. (2026) and Černis et al. (2026) provide important foundational knowledge that in adolescence (a) sense of anomaly is normatively elevated and (b) self‐reports begin to reflect distinct experiences of psychopathology that may not be adequately captured by other informants. Unsurprisingly, as the adolescent experience becomes more complex, the body and brain are challenged to adapt to changing environments and novel experiences. The exciting new findings from Martinez et al. (2026) and Černis et al. (2026) scaffold progress toward two critical questions in the field: (1) how does true body change, such as during puberty, alter anomalous experience risk and (2) through which biopsychosocial pathways might atypical anomalous experiences drive psychopathology over time?

Beginning to answer these questions, recent work from our group used a body mapping measure to assess self‐reported experiences of body anomaly, prioritizing spatial accuracy (Moussa‐Tooks et al., 2026). Consistent with Černis et al. (2026), adult participants retrospectively reported expected changes (i.e., “feels new, strange, unfamiliar”) during puberty. This included regions such as breasts, hips, face, groin, and biceps. Importantly, for some individuals, these experiences persisted to adulthood. Using the ČEFSA, we found that anomalous experiences of self, body, and agency were associated with greater persistence of bodily change on the self‐report maps (Moussa‐Tooks et al., 2026). Although disturbances in agency appeared to be normative, anomalous experience of self and body are rarer per Černis et al. (2026), likely indicating higher psychopathology risk. Aligned with this prediction, we found that anomalous body experiences that persist beyond puberty and extend beyond expected physical areas of change are associated with more psychotic‐like experiences (Moussa‐Tooks et al., 2026). This was not the case for depression or anxiety, highlighting that these disturbances may reflect distinct mechanisms. Puberty, and other reproductive transitions, increase vulnerability when changing bodily signals cannot be accurately predicted, localized, or integrated. Compromised ability to process body signals increases the likelihood that ordinary sensations are experienced as unusual, externally generated, or personally significant. It is likely that aberrant body experiences serve as process‐level mediators of biopsychosocial change. That is, hormone and brain‐based changes drive alterations in sensorimotor systems, that then give rise to the experience of anomaly. In turn, distinct anomalous experiences drive unique phenotypes that, when persistent, become disordered (e.g., dissociation, psychosis, eating and feeding disorders, chronic pain etc.) (Sestito et al., 2017).

Future work should take a more comprehensive biopsychosocial approach. Sex hormones have a variety of targets, including in the brain, that drive changes in behavior and perception spanning the spectrum of normative to aberrant. In fact, prior work from our group suggests that sex hormones associated with adrenarche, like dehydroepiandrosterone, may drive the emergence of psychotic‐like experiences (Chaku & Barry, 2024; Larson et al., 2026). Ovarian hormones similarly cross the blood‐brain barrier where they act on neural circuits that are densely expressed in the prefrontal cortex, hippocampus, and cerebellum, among other regions. Through these mechanisms, sex hormones modulate the development of the brain and body, highlighting a parsimonious and scientifically useful biochemical explanation for the onset of psychopathology during adolescence. These biological changes unfold within a dynamic social context. For example, our prior work lends support to the maturational deviance model, by which social deviance from peers likely contributes to puberty‐related differences in psychosis risk (Larson et al., 2026). Hormonal changes may therefore shape psychopathology not only through their direct effects on neural systems, but also through changes in peer experiences, body image, emotion regulation, and interpretations of the self and others. Consistent with this possibility, Černis et al. (2026) report that expressive suppression mediates the relationship between felt anomaly across a 1‐month timespan. It will be critical to disentangle how these perceptual and cognitive processes interact and unfold over time, especially during the adolescent period that provides a natural experiment of body change.

There are several additional compelling future directions to consider. As this area of inquiry progresses, longitudinal studies are necessary to capture change over time and better characterize normative developmental trajectories from the aberrant trajectories that suggest risk or resilience. Longitudinal studies should consider atypical versus typical development, multiple types of physical transitions (i.e., pregnancy, menopause), and the importance of sex differences, and cultural diversity given the various ways in which bodily changes are perceived, interpreted, and celebrated. Greater attention to these individual and contextual differences may also clarify when and why youth‐ and co‐informant reports diverge, as such discrepancies may vary systematically across sociodemographic characteristics and risk factors rather than representing uniform differences in reporting (Martinez et al., 2026).

In sum, biopsychosocial mechanisms emerge and transform across adolescence and young adulthood. Changes rooted in the body and one's body perception appear to be essential to linking hormonal, brain, and social mechanisms to psychopathology and understanding the complex etiology underlying the emergence of clinical disorders. The new work described here from Martinez et al. (2026) and Černis et al. (2026) provide important foundation to understand how anomalous experiences serve as transdiagnostic markers for such risk.

## AUTHOR CONTRIBUTIONS

**Alexandra Moussa‐Tooks**: Conceptualization; writing—original draft; writing—review and editing. **Natasha Chaku**: Conceptualization; writing—original draft; writing—review and editing.

## CONFLICT OF INTEREST STATEMENT

The authors declare no conflicts of interest.

## ETHICAL CONSIDERATIONS

Ethics requirements do not apply to this Commentary article.

## AI STATEMENT

The Authors declare that they have not used AI in any capacity that would require disclosure in accordance with Wiley's AI Guidelines, nor in any capacity that would reasonably require discloser for editors, reviewers and readers to properly evaluate their research or manuscript. The authors confirm that AI has not been used for purposes including but NOT limited to drafting and editing, to generate substantial text or restructure arguments, nor in the research methodology. The authors take full responsibility for the accuracy of this statement.

## Data Availability

No data are presented in this commentary; data for the empirical papers is described in their respective publications.

## References

[jcv270166-bib-0001] Černis, E. , Antonovic, M. , Lofthouse, K. , Shipp, L. , & Waite, P. (2026). ‘Felt sense of anomaly’‐type transdiagnostic dissociative experiences in adolescents: Endorsed phenomenology and plausible mechanisms. JCPP Advances, e70116. 10.1002/jcv2.70116 42416669 PMC13339579

[jcv270166-bib-0002] Chaku, N. , & Barry, K. (2024). Exploring profiles of hormone exposure: Associations with cognition in a population‐based cohort of early adolescents. Infant and Child Development, 33(2), e2415. 10.1002/icd.2415

[jcv270166-bib-0003] Chaku, N. , & Davis‐Kean, P. E. (2024). Positioning adolescence in the developmental timeline. Journal of Research on Adolescence, 34(4), 1191–1200. 38752795 10.1111/jora.12928 PMC11606252

[jcv270166-bib-0004] Larson, E. R. , Chaku, N. , & Moussa‐Tooks, A. (2026). Early pubertal development is a risk factor for psychotic‐like experiences in boys and girls. Biological Psychiatry Global Open Science, 6(2), 100647. 10.1016/j.bpsgos.2025.100647 41542154 PMC12799914

[jcv270166-bib-0005] Martinez, M. , Mittal, V. A. , Schaefer, J. D. , Haase, C. M. , & Damme, K. S. F. (2026). Diverging perspectives on emerging mental health symptoms: Multi‐informant discrepancies and their associated determinants across the transition to adolescence in the Adolescent Brain Cognitive Development Study. JCPP Advances, e70122. 10.1002/jcv2.70122 42416656 PMC13339503

[jcv270166-bib-0006] Moussa‐Tooks, A. , Eribo, M. , & Chaku, N. (2026). Self‐report mapping of puberty‐based anomalous body experience [Preprint]. PsyArXiv.

[jcv270166-bib-0009] Paster, G. K. (1993). The body embarrassed: Drama and the disciplines of shame in early modern England. Cornell University Press. ISBN: 978-1-5017-2449-7.

[jcv270166-bib-0007] Petersen, M. W. , Schröder, A. , Jørgensen, T. , Ørnbøl, E. , Meinertz Dantoft, T. , Eliasen, M. , Benros, M. E. , & Fink, P. (2020). Irritable bowel, chronic widespread pain, chronic fatigue and related syndromes are prevalent and highly overlapping in the general population: DanFunD. Scientific Reports, 10(1), 3273. 10.1038/s41598-020-60318-6 32094442 PMC7039919

[jcv270166-bib-0008] Sestito, M. , Raballo, A. , Stanghellini, G. , & Gallese, V. (2017). Editorial: Embodying the self: Neurophysiological perspectives on the psychopathology of anomalous bodily experiences. Frontiers in Human Neuroscience, 11, 631. 10.3389/fnhum.2017.00631 29311881 PMC5742196

